# Short-term deleterious effects of standard isolation and cultivation methods on new tropical freshwater microalgae strains

**DOI:** 10.7717/peerj.5143

**Published:** 2018-07-19

**Authors:** M. Magdalena Aray-Andrade, Miguel I. Uyaguari-Diaz, J. Rafael Bermúdez

**Affiliations:** 1 ESPOL Polytechnic University, Escuela Superior Politécnica del Litoral, ESPOL, Plankton Laboratory, Facultad de Ingenería Marítima, Ciencias Biológicas, Oceánicas y Recursos Naturales, Campus Gustavo Galindo, Guayaquil, Ecuador; 2 Joint Postgraduate VLIR NETWORK Master Program in Applied Biosciences, Biodiscovery, ESPOL Polytechnic University, Guayaquil, Guayas, Ecuador; 3 Escuela de Medicina, Universidad Espíritu Santo-Ecuador, Samborondón, Guayas, Ecuador; 4 British Columbia Centre for Disease Control Public Health Laboratory, Vancouver, BC, Canada; 5 Department of Pathology and Laboratory Medicine, University of British Columbia, Vancouver, BC, Canada; 6 Galapagos Marine Research and Exploration, GMaRE. Joint ESPOL-CDF program, Charles Darwin Research Station, Galapagos Islands, Ecuador

**Keywords:** Lipids, Growth rate, Antibiotics, Cryopreservation, Microalgae

## Abstract

Algae with potential biotechnological applications in different industries are commonly isolated from the environment in order to obtain pure (axenic) stocks that can be safely stored for long periods of time. To obtain axenic cultures, antibiotics are frequently employed, and cryopreservation is applied to preserve standing stocks. However, many of these now standard methods were developed using strains derived from pristine to near-pristine environments and cold to temperate regions. The potential effect of the said methods on the life cycle and biochemical profile of algae isolates from hyper-eutrophic and constant high-temperature tropical regions is not well understood. These effects could potentially render them unsuitable for their intended biotechnological application. In this study, we conducted a genetic characterization (18S rRNA) and evaluated the effect of purification (the use of the antibiotic chloramphenicol, CAP) and cryopreservation (dimethyl sulfoxide; DMSO–sucrose mix and glycerol) on the growth rate and lipid content of three new tropical freshwater algal isolates: *Chorella* sp. M2, *Chlorella* sp. M6, and *Scenedesmus* sp. R3, obtained from the Ecuadorian coast. The genetic and morphological characterization revealed a clear discrimination between these strains. All strains cultured with CAP exhibited a lower growth rate. Subsequent to cryopreservation, *Chorella* sp. M2, *Chlorella* sp. M6, and *Scenedesmus* sp. R3 presented no significant difference in growth rate between the cryopreservants. Further, a significantly higher lipid content was observed in the biomass cryopreserved with glycerol in relation to the DMSO–sucrose, with *Chorella* sp. M2 and *Chlorella* sp. M6 having twice as much as they had in the first treatment. These results highlight the relevance of selecting an appropriate method for storage, as the materials used can affect the biological performance of different tropical species, although it is still to be determined if the effects observed in this study are long lasting in subsequent cultures of these algae.

## Introduction

The cultivation of wild microalgae under defined/controlled conditions has received considerable interest due to their potential as sources of various compounds that have biotechnological and commercial for the nutraceutical, cosmetic, and pharmaceutical industries (Dogaris et al., 2015; Encarnação et al., 2015; Gellenbeck, 2012; Maurya et al., 2016; Rodrigues et al., 2015). Some microalgae serve as essential raw materials in biofuel production due to their ability to accumulate a high lipid content. Microalgae such as *Chlorococcum* sp. represents a fast-growing specie (John et al., 2011; Mahapatra & Ramachandra, 2013; Masojídek et al., 2000), and its lipid content can contribute up to 30.55 ± 2.65% of dried biomass (Mahapatra & Ramachandra, 2013). Some *Chlorella* species are known to produce lipids such that 10–39% of dried biomass is lipids (Chiu et al., 2015), showing a huge potential as a lipid sources in biotechnology. Recently, the production of microalgal biomass has been scaled up to large outdoor cultivation systems for the production of biodiesel. High temperatures can severely affect the productivity of these systems (Béchet et al., 2013). The isolation and characterization of high-temperature-tolerant microalgal species is important for mitigating these effects, as has been demonstrated with the temperature-tolerant strain *Chlorella sorokiniana* (Béchet et al., 2013).

One advantage of industrial algal biomass production is that wastewater can be used as a culture media to produce middle- and high-value by-products. The use of wastewater reduces the use of freshwater, decreases the cost of nutrient addition, and assists in the removal of nitrogen and phosphorus, which otherwise would have been released into the environment (Chiu et al., 2015). Several microalgae have been shown to have high bioremediation potential, such as *Chlorella* and *Scenedesmus* (Silkina et al., 2017). A significant barrier to using wastewater is that it has such high levels of nitrogen, phosphorus, and other microbes, that many microalgal species have their growth inhibited by it (Mahapatra & Ramachandra, 2013; Mara, 2004). An efficient strategy to mitigate the potential negative effects of using wastewater as culture media is the isolation of wild algal strains proliferating in it (Mahapatra & Ramachandra, 2013). However, to assess the biotechnological potential and applications of microalgal species, the first step in assessing the biotechnological potential of microalgal species is to isolate them from the environment and obtain pure (axenic) stocks that can be stored for long periods of time and reused. Given that an elevated bacterial contamination can be expected during the isolation of fresh strains from wastewater (Mara, 2004), antibiotics such as florfenicol, streptomycin, furazolidone, and specially chloramphenicol (CAP) are often used to eliminate bacteria in the culture media (Campa-Córdova et al., 2006; Lai et al., 2009). CAP is highly effective because it inhibits a variety of aerobic and anaerobic microorganisms. However, intensive use of antibiotics in households, agriculture, and aquaculture has shown adverse ecological effects, including the development of resistant bacterial populations (Lai et al., 2009), which may require a higher concentration of antibiotics for their elimination from isolated strains. It has been reported that antibiotics can negatively affect the growth of certain algal species, such as *Chlorella pyrenoidosa*, *Isochrysis galbana*, and *Tetraselmis chui* (Lai et al., 2009) which are cultured with CAP.

The cryopreservation of microalgae stocks for long-term storage is important, as it eliminates the requirement to maintain continuous batches of growing strains (Bui et al., 2013). Although the mechanisms involved in cryopreservation are generally understood, the impact is not (Bui et al., 2013) especially on difficult-to-freeze algae isolated from tropical environments (Fuller, Lane & Benson, 2005). Cryopreservation can cause the formation of ice crystals in the cellular cytoplasm during the freezing process, which disrupt the cell membrane (Bui et al., 2013). In order to reduce this effect, cryopreservants can be added to the culture media to protect eukaryotic cells from the damage caused by freezing. For instance, dimethyl sulfoxide (DMSO), methanol, and glycerol are cell-wall-permeable cryoprotectants. Another cryoprotectant, sucrose, does not permeate the cell membrane and thus its protection is extracellular (Bui et al., 2013; Hubálek, 2003). While the cryopreservant may protect the cell, it also may change the original biochemical profile of the algae through the alteration of their genetic structure and/or metabolic pathways (Müller et al., 2007), making them unsuitable for their intended biotechnological application, for which they were originally isolated.

A crucial step in the potential use of tropical algae as a biotechnological resource is determining the potential effects of commonly employed methods on their biochemical profiles and productivity rates. Thus, the aim of the present study was to evaluate the effect of traditional isolation and cultivation processes used to obtain axenic cultures (treatment with chloramphenicol) and their cryopreservation (DMSO–sucrose and glycerol) on the growth rate and lipid content of four new algae isolates obtained from wastewater in the tropical Ecuadorian coast region.

## Materials and Methods

### Isolation, identification, and preparation of stocks

For this study, microalgae strains were isolated from green standing water bodies (20 ml) located around the urban areas of Guayaquil, Ecuador. From each water sample collected, serial 1:10 dilutions were made in 1.5 ml eppendorf tubes using Guillard’s f/2 medium (Guillard & Ryther, 1962) as the culture media. The medium was made using filtered freshwater instead of saltwater.

The strains, identified with morphological differentiation, were as follows: *Chorella* sp. M2, *Chlorella* sp. M6, and *Scenedesmus* sp. R3; Wehr & Sheath’s (2003) method was followed.

The isolated and identified strains were further cultivated in batch cultures with Guillard’s f/2 medium, under controlled laboratory conditions (culture cabins at 30 °C and a 12/12 h photoperiod), in order to obtain sufficient biomass to start the experiments.

### Antibiotic assays

In order to obtain an axenic culture, CAP was added to the culture media at a final concentration of 50 mg l^−1^, as recommended by Lai et al. (2009). This concentration was selected because the strains were isolated from hypereutrophic stagnant ponds located around urban areas of Guayaquil; therefore, they were expected to contain an elevated bacterial load.

The experiment to determine the effect of the antibiotic on growth rate comprised three 48-h phases: (1) before the addition of antibiotics, (2) with antibiotics, and (3) after the removal of antibiotics. The removal was done by replacing the culture media with another culture media without using an antibiotic three times in order to rinse off the remnants. Microalgae strains were cultivated in Guillard’s f/2 medium in triplicates on a semi-continuous batch set up over 6 days at 30 °C and a photoperiod of 12:12. After every growth phase, new culture media were supplied to each bottle to eliminate the bottle effect.

The cell number of each culture, expressed as cells per milliliter (IOC-UNESCO, 2010), was determined after each phase of the experiment using a Neubauer chamber and compound microscope. The growth rate, expressed as µ, was determined by calculating the counting difference between the last and first day of culture divided by the total number of days required by the culture; further, the growth rates of each phase of the experiment were compared.

### DNA extraction, polymerase chain reaction, cloning, and sequencing

After obtaining the axenic cultures, the algae were genetically characterized. Before extraction, to facilitate the disruption of algal cells, eight freeze-thaw cycles were conducted, followed by overnight digestion, using 20 μl of 20 mg ml^−1^ proteinase K (Qiagen Sciences, Germantown, MD, USA). The DNA was extracted using the UltraClean Soil DNA Kit (MoBio, Carlsbad, CA, USA) following the manufacturer’s instructions. The DNA concentration and purity were assessed with Qubit dsDNA high sensitivity in a Qubit 3.0 fluorometer (Life Technologies, Carlsbad, CA, USA) and NanoDrop spectrophotometer (NanoDrop technologies, Inc., Wilmington, DE, USA) respectively. Specific primers targeting V1–V3 regions of the 18S rRNA gene were selected based on previous studies (Amann, Krumholz & Stahl, 1990; Zhu et al., 2005; Amann, Krumholz & Stahl, 1990; Zhu et al., 2005). Each 50 μl of PCR reaction consisted of 1.5 mM MgCl2, 0.2 mM nucleotides, 400 mM of each primer, 1.25 U of hot start polymerase (Promega Corporation, Fitchburg, WI, USA), and one μl of template DNA. The thermal cycling conditions entailed 94 °C × 5 min, 35 cycles of 30 s at 94 °C, 60 s at 55 °C, and 90 s at 72°C, and a final cycle of 10 min at 72 °C. PCR amplicons were examined in a 1.5% agarose/0.5× TBE gel stained with 1× GelRed (Biotium, Inc., Hayward, CA, USA) and purified with a QIAQuick PCR Purification Kit (Qiagen Sciences, Maryland, MD, USA) according to the manufacturer’s instructions. Subsequently, the amplicons were ligated into pCR2.1-TOPO cloning vectors (Invitrogen, Carlsbad, CA, USA) and transformed into One Shot *E. coli* DH5 α-T1R competent cells following the manufacturer’s protocol. At least four transformants per sample were screened for inserts following the manufacturer’s recommendations. Positive transformants were subsequently grown overnight at 37 °C in a lysogeny broth with 50 μg ml^−1^ kanamycin. Plasmids were extracted using the PureLink Quick Plasmid Miniprep Kit (Life Technologies, Carlsbad, CA, USA). A dsDNA high sensitivity kit in a Qubit 3.0 fluorometer was employed for further quantitation assessment (Life Technologies, Carlsbad, CA, USA). Plasmids were end-sequenced in an ABI 3130xl Genetic Analyzer (Life Technologies, Carlsbad, CA, USA) with a M13 forward primer (−20) (5′-GTAAAACGACGGCCAG-3′) and M13 reverse primer (5′-CAGGAAACAGCTATGACC-3′) using BigDye Terminator version 3.1 cycle sequencing kit (Applied Biosystems, Warrington, UK). The resultant set of DNA sequences were blasted against the SILVA 18S rRNA database (Pruesse et al., 2007).

The partial sequences were deposited into GenBank *Chlorella* sp. M2 (accession number: MF677855), *Chlorella* sp. M6 (accession number: MF677856), *Scenedesmus* sp. R3 (accession number: MF677857).

### Phylogenetic tree construction

To further define the sequences in this study, the sequence alignment of 18S rRNA gene was performed with MAFFT multiple aligner and the tree constructed with Jukes–Cantor (Genetic distance model); Method employed: UPGMA with 1,000 iterations (Kearse et al., 2012).

### Cryopreservation assays

In order to determine the effect of cryopreservation on the growth rate and lipid content of the new tropical algal isolates, they were subjected to two treatments, set in triplicates, consisting of glycerol and a DMSO–sucrose mix, respectively. The cultures of *Chorella* sp. M2, *Chlorella* sp. M6, and *Scenedesmus* sp. R3 with concentration in the range of 2–9 × 10^7^ ml^−1^ cells were used (Bui et al., 2013). One milliliter of the culture was transferred to 1.8 ml cryovials for the respective treatments. A DMSO and sucrose solution (Fisher Chemical, CSA grade) was added to a final concentration of 10% (v/v) and 200 mM (v/v), respectively (Bui et al., 2013), while glycerol was added to a final concentration of 10% (v/v) (Hubálek, 2003) in the corresponding vials. The cryopreservants were added slowly to the cryovials containing the algae as recommended by Bui et al. (2013). Additionally, a negative control comprising cryovials of each microalgae strain without cryopreservants be was prepared.

The cryovials were inserted into a polyethylene container with a screw cap filled with 125 ml isopropanol (Bui et al., 2013). Isopropanol enables a freezing rate of approximately −1 °C min^−1^ (Shiraishi, 2016). This container was stored in a freezer at −80 °C for short (5 days) and long (14 months) periods, respectively, in order to compare the effect of cryopreservation on time growth rate of the algal; the short period was considered because it represents the approximate time required for the replenishment of stocks in semi-continuous cultures with harvest every 5 days; the second was considered to evaluate the storage of stocks for long durations. At the end of each period, the container was removed from the freezer and the cryovials were put to float in a beaker filled with water (∼500 ml at 25 °C) (Bui et al., 2013), four cryovials at a time. The thawed cell cultures were gently poured into sterile Falcon tubes containing 49 ml of Guillard’s f/2 medium at room temperature; it was left unmixed for one h and subsequently carefully inverted five times (Bui et al., 2013). To allow cell recovery after thawing and before the experiments started, the Falcon tubes with the strains were kept at 30 °C in the dark for 24 h, at dimmed-light for 48 h and with light with a photoperiod of 12:12 again for 48 h. After this adaptation period, the cultures were transferred to glass vials where they were kept in batch cultures for 6 days before the experiment started. For the experiment, the microalgae were cultivated at 30 °C and a photoperiod 12:12 for 6 days. At the end of the culture period, the growth rates, expressed as µ, was determined by calculating the counting difference between the last (day 6) and day 1, and sample for algal lipid content were taken by filtering a volume of 10 ml from each culture using Whatman GF/F and GF/D glass fiber filters (∼0.7 and 2.7 μm pore size, respectively) in accordance to the size of the microalgae.

### Total lipid

The total lipid content from the cryopreservation test was determined following the protocol described by Christie (1994), using a mix of chloroform, dichloromethane, and methanol (1:1:1). The ethereal extract was determined in terms of weight difference (Matos et al., 2016; Prommuak et al., 2012; Yang et al., 2014).

### Statistical analysis

The statistical analysis was performed with analysis of variance within (one-way ANOVA) and between strains (two-way ANOVA) using the Statistica v7.0.61.0 software. The normality of distribution was analyzed with a Shapiro test. A *p*-value of < 0.05 was considered as statistically significant. Furthermore, a post hoc analysis was conducted with a Tukey test in order to determine the significance. The total lipid was analyzed with nonparametric Kruskal–Wallis and median tests, as the data revealed a non-normal distribution.

## Results and Discussion

**Figure 1:**
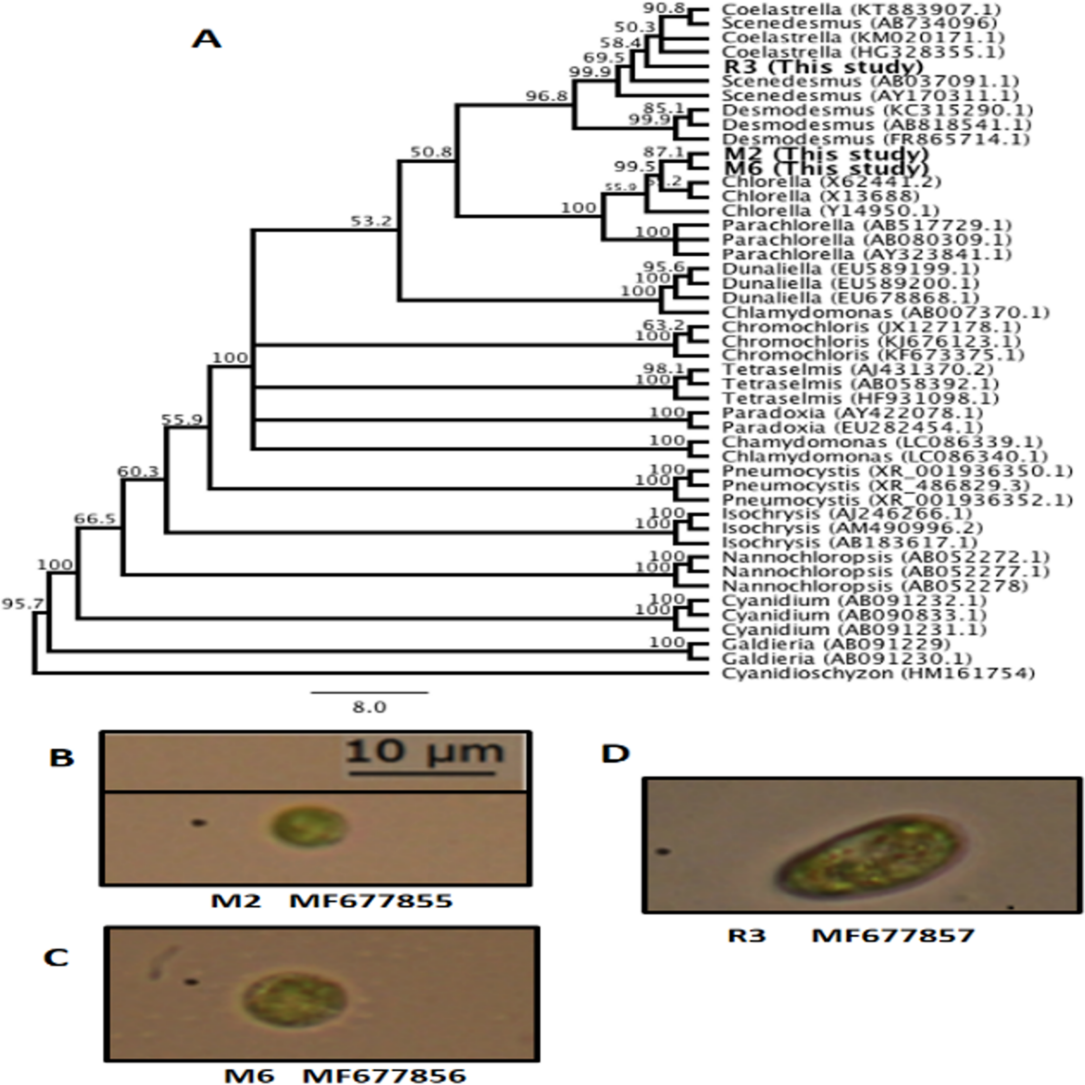
Microalgae isolated in this study. (A) Unweighted pair group method with arithmetic mean (UPGMA) consensus tree of the 18S rRNA sequences isolated in this study and other representative algal sequences retrieved from the National Center for Biotechnology Information (NCBI) database. Numbers in the branches represent bootstrap values from 1,000 replications. Numbers in parentheses are the GenBank accession identification numbers. (B) *Chlorella* sp. M2 MF677855. (C) *Chlorella* sp. M6 MF677856. (D) *Scenedesmus* sp. R3 MF677857.

The genetic identification conducted with 18S rRNA partial sequences and the SILVA database corresponded with the morphological identification, with the most probable genera being *Chorella* sp. M2, *Chlorella* sp. M6, *Scenedesmus* sp. R3. Moreover, to determine the possible association between our sequences and other characterized algal sequences in the GenBank database, an UPGMA consensus tree was created (Fig. 1A). The consensus sequence percentage revealed a 90% similarity between M2 and M6 in nucleotide alignment. Sequences from M2 and M6 exhibited a 67.9% similarity with members of the genera *Chlorella*. Overall, M2 and M6 clustered with members of the Chlorellaceae family and included genera such as *Chlorella* and *Parachlorella*. These results indicate the relationship between members of this family as previously reported (Bock, Pröschold & Krienitz, 2011; Krienitz et al., 2004). R3 clustered with members of the Scenedesmaceae family, including *Coelastrella*, *Scenedesmus*, and *Desmodesmus* (Fig. 1A). The small discrepancies observed in the definition of genera in the clades may be caused due to the partial sequences obtained in this study. Nevertheless, bootstrap values were found to be higher than 50% for these branches. These findings are also supported by the morphological identification conducted following Wehr & Sheath’s (2003) method for all algal isolates from this study, *Chlorella*. M2 (Fig. 1B), *Chlorella*. M6 (Fig. 1C), and *Scenedesmus* R3 (Fig. 1D).

### Growth rate using chloramphenicol

**Figure 2:**
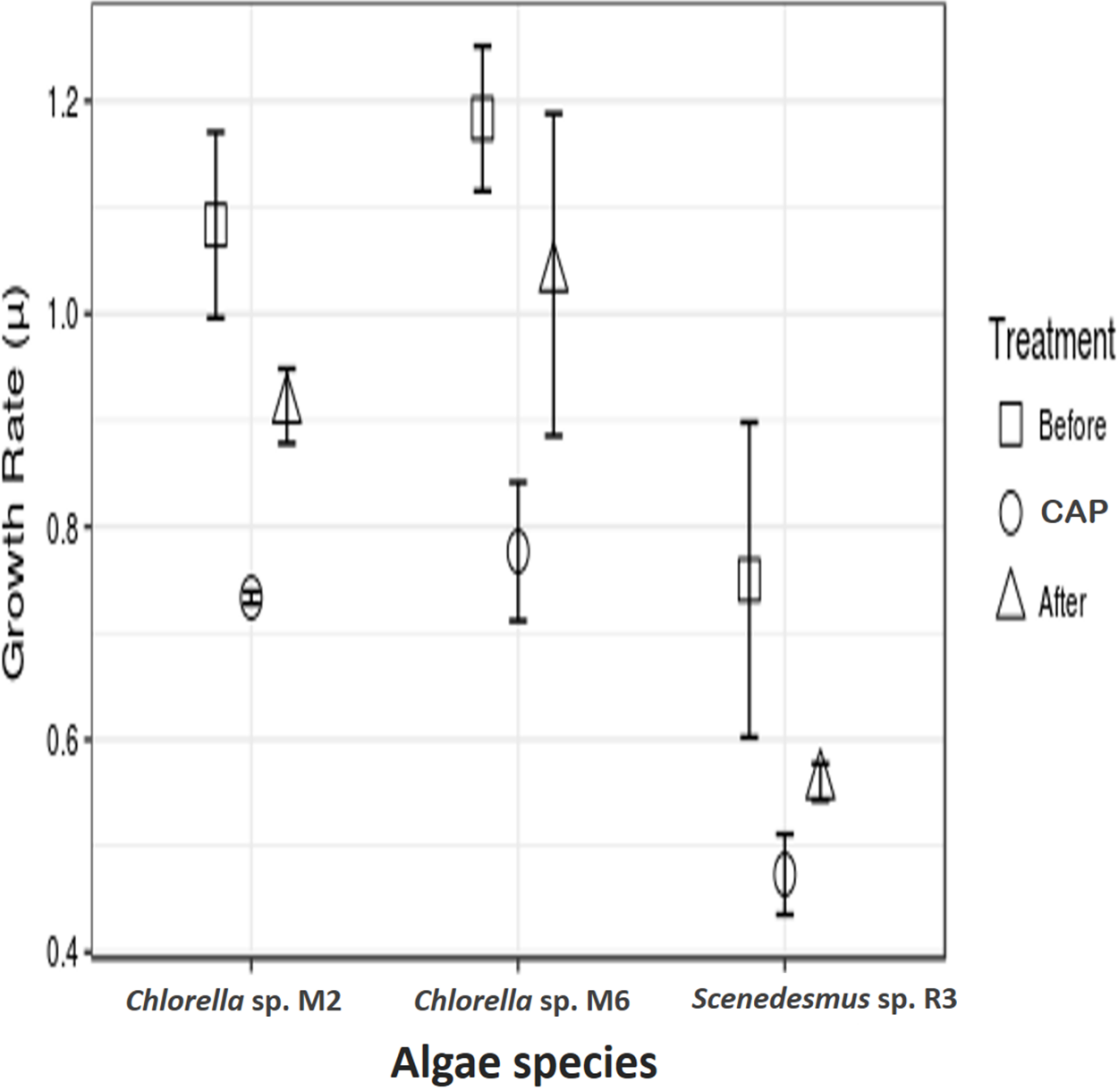
Antibiotic assays. Growth rate of microalgae before the addition of antibiotics, with chloramphenicol in final concentration of 50 mg l^−1^ (CAP) and after the removal of antibiotics.

The microalgae revealed significant differences in growth rate with and without CAP across them (ANOVA, *F* = 47.99, *p* = 0.0002, d*f* = 2; post hoc in Table 1) and also within each strain (ANOVA, *Chlorella* sp. M2: *F* = 47.94, *p* = 0.00228; *Chlorella* sp. M6: *F* = 55.96, *p* = 0.0017; *Scenedesmus* sp. R3: *F* = 9.84, *p* = 0.034945; post hoc in Table 2) (Fig. 2). Similar results have been reported by Lai et al. (2009), who found concentrations 20–40 mg l^−1^ of CAP inhibited growth in *C. pyrenoidosa*, which is close to the range employed in the present study (50 mg l^−1^). This effect has been attributed to the CAP acting as an inhibitor of photosynthetic oxygen evolution as well as an inhibitor of protein synthesis in chloroplasts, that, in turn, affects the chlorophyll synthesis in photosynthetic organisms. Seoane et al. (2014) reported a decrease in cell size and in chlorophyll *a* content but detected an increase in chlorophyll *a* fluorescence in microalga *Tetraselmis suecica*. This could be due to an inhibitory effect localized on the oxidant side of mitochondria, caused by blockage of the electron transport chain at the photosystem (PS) II level. The inhibition of the electron flow in the PS II reaction center at the donor side provokes a decrease in the chlorophyll *a* fluorescence, while if the inhibition is produced in the acceptor side of the PS II, an increase in the chlorophyll *a* fluorescence is observed (Cid et al., 1995).

**Table 1:** Result of the post hoc statistical analysis of antibiotic treatments.

Microalgae	Before CAP	Addition CAP	Removal CAP	DMSO + sucrose	Gly	Without cryopreservants
*Chlorella* sp. M2–*Chlorella* sp. M6	0.522912	0.486532	0.289684	**0.000721**	**0.028756**	0.103303
*Chlorella* sp. M2–*Scenesdesmus* sp. R3	**0.020487**	**0.001001**	**0.007372**	**0.001287**	**0.028756**	**0.002993**
*Chlorella* sp. M6–*Scenesdesmus* sp. R3	**0.006173**	**0.000535**	**0.001764**	0.623298	1.00000	**0.038330**

**Notes:**

Before, addition and removal of chloramphenicol (CAP); cryopreservation treatments with dimethyl sulfoxide + sucrose (DMSO + S), glycerol (Gly) and without cryopreservants. All microalgae combinations are listed. The values given are *p* values. Values with *p* < 0.05 are presented in bold.

**Table 2:** Result of the post hoc statistical analysis of antibiotic treatments.

	Before CAP	Addition CAP	Remove CAP	DMSO–Sucrose	Glycerol	Without cryopreservants
***Chlorella* sp. M2**						
Before CAP		**0.000725**	**0.020553**			
Addition CAP	**0.000725**		**0.016004**			
Remove CAP	**0.020553**	**0.016004**				
DMSO–sucrose					0.0728360	**0.015240**
Glycerol				0.0728360		**0.037254**
Without cryopreservants				**0.015240**	**0.037254**	
***Chlorella* sp. M6**						
Before CAP		**0.0007049**	0.265358			
Addition CAP	**0.0007049**		**0.048635**			
Remove CAP	0.265358	**0.048635**				
DMSO–sucrose					0.102721	**0.000430**
Glycerol				0.102721		**0.001537**
Without cryopreservants				**0.000430**	**0.001537**	
***Scenedesmus* sp. R3**						
Before CAP		**0.020667**	0.087554			
Addition CAP	**0.020667**		0.497653			
Remove CAP	0.087554	0.497653				
DMSO–sucrose					0.507627	0.084745
Glycerol				0.507627		0.367752
Without cryopreservants				0.084745	0.367752	

**Notes:**

Before, addition and removal of chloramphenicol (CAP); cryopreservation treatments with dimethyl sulfoxide + sucrose (DMSO + S), glycerol (Gly), and without cryopreservants. The values given are *p* values. Values with *p* < 0.05 are presented in bold.

In addition to the toxic effect of CAP on photosynthesis, the high antibiotic concentration used in the present study eliminated the possibility of the presence of bacteria associated with freshly isolated strains, which could have contributed to the observed negative effect. It is known that microalgae live in synergism with certain bacteria, interacting with each other through nutrients interchange, signal transduction, genes transference, and other means (Kouzuma & Watanabe, 2015). Lubarsky et al. (2010) demonstrated that the microalgal biomass was significantly lower in axenic cultures (the antibiotics added) as compared to the cultures containing bacteria. Nevertheless, the two strains of *Chlorella* demonstrated a higher growth rate in relation to *Scenedesmus* sp. after the removal of CAP. This demonstrates that some microalgae strains can recover from CAP exposure, such as *Chlorella* sp. M2 and M6, result observed here and also by other authors, such as Campa-Córdova et al. (2006) and Lai et al. (2009), who employed CAP at concentrations of 0.5 × 12.0 mg l^−1^. CAP has been shown to have no significant effect in the growth of *C. pyrenoidosa* and *I. galbana*. A more detailed study is necessary to access the actual cause of the observed effect.

### Cryopreservation test

**Figure 3:**
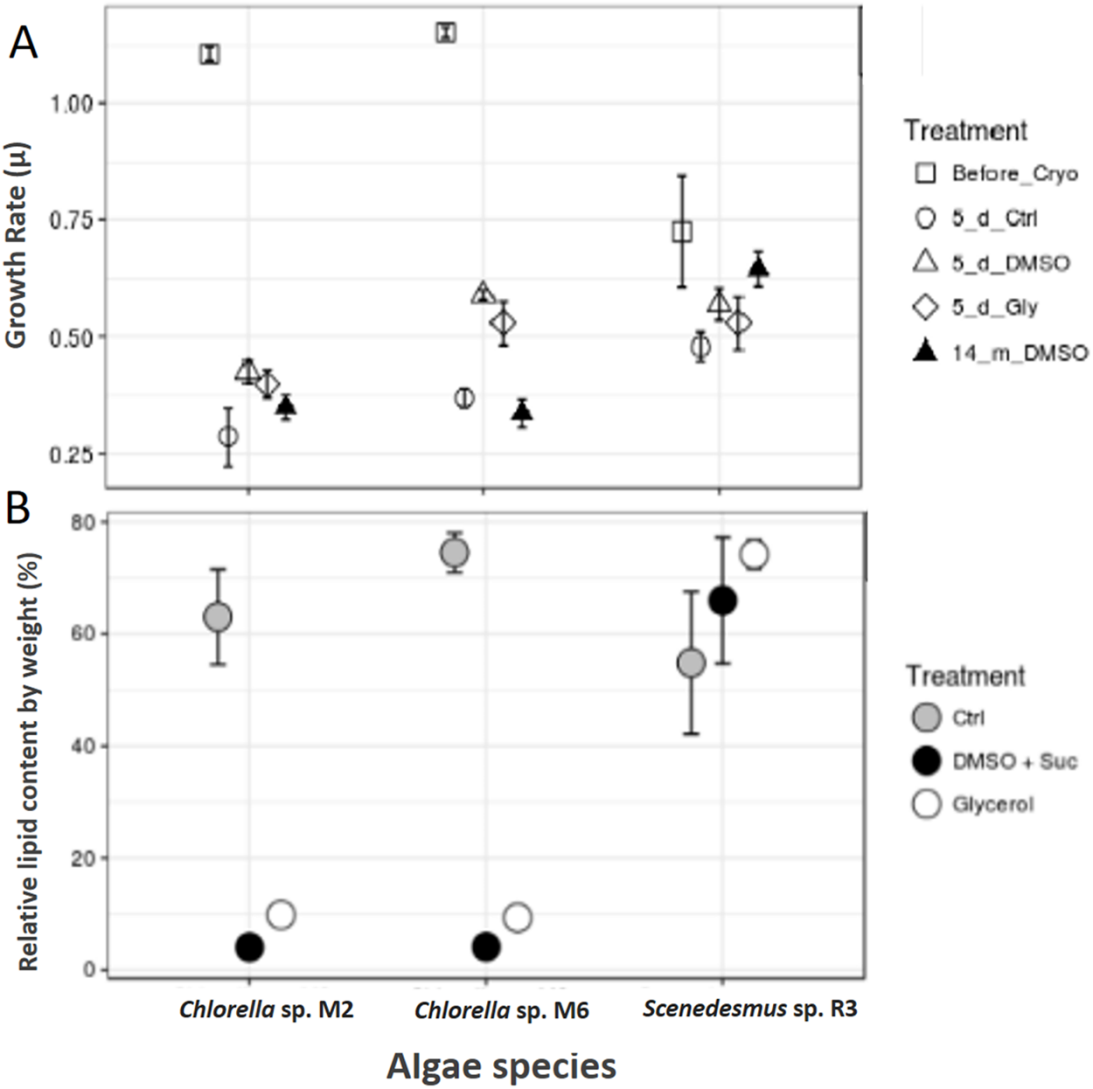
Cryopreservation test and total lipid. (A) Microalgae growth rate before and after cryopreservation during 5 days with dimethyl sulfoxide and sucrose (DMSO), glycerol (Gly), plus a control without cryopreservant (Ctrl) and after cryopreservation during 14 months with dimethyl sulfoxide and sucrose. (B) Total lipids of microalgae, control (Ctrl), after cryopreservation with dimethyl sulfoxide (DMSO + sucrose) and glycerol.

*Scenedesmus* sp. R3 did not show a difference in its growth rate before and after cryopreservation, unlike the two other strains (ANOVA, *F* = 3.52, *p* 0.097448; post hoc in Table 2) (Fig. 3A). Saadaoui et al. (2016) reported that *Scenedesmus* cells rapidly recover after 1 month of storage in liquid nitrogen using dimethyl sulfate as cryoprotectant. Microalgae exhibited differences in growth rate among different strains (ANOVA, *F* = 925.02, *p* < 0.001, d*f* = 3; post hoc in Table 1). Our two strains of *Chlorella* sp. after 14 months of cryopreservation showed a growth rate lower than the strain cryopreserved for 5 days. No microalgal strains showed different growth rates between cryopreservation types; however, the cultures that were frozen without a cryopreservant demonstrated a lower growth rate compared to cultures with cryopreservants; they also required more time for recovery (ANOVA, *Chlorella* sp. M2: *F* = 9.44, *p* 0.014015; *Chlorella* sp. M6: *F* = 44.77, *p* 0.000248; post hoc in Table 2) (Fig. 3A). This may be due to the need for permeable and impermeable cryopreservants such as DMSO–sucrose, because sucrose increases solution osmolarity that causes cell dehydration. Sucrose works synergistically with DMSO to minimize intracellular ice damage by increasing the total concentration of the solute and reducing the amount of ice formed in the cell (Bui et al., 2013). Moreover, glycerol decreases the freezing point of water and biological fluids through colligative action (glycerol/water to a minimum of –46 °C), and it prevents eutectic crystallization (Hubálek, 2003). *Scenedesmus* sp. R3 exhibited the smallest difference between the growth rates of cultures with cryopreservation and without cryopreservants. A more detailed study is necessary to evaluate the effect of frostbite without cryopreservants.

### Total lipid content

The total lipid content was analyzed in the biomass of microalgae strain using cryopreservants. *Scenedesmus* sp. R3 showed an increase in the total lipid content after cryopreservation, unlike the other two strains that showed a decrease by a large proportion (Chi square = 5.955556, d*f* = 2, *p* = 0.0509) (Fig. 3B). Saadaoui et al. (2016) confirmed that the lipid production could be affected by the cryopreservation method that could result in an increase and decrease in lipid content. When the two cryopreservants were compared, a higher lipid content was observed in the biomass that was cryopreserved with glycerol. Thus, in *Chorella* sp. M2 and *Chorella* sp. M6, the relative lipid content was twice as high compared to the biomass cryopreserved with DMSO + sucrose (Chi-square = 6.300000, d*f* = 2, *p* = 0.0429) (Fig. 3B). This might be due to the fact that some algae can utilize glycerol as a carbon source. Since, *Chlorella vulgaris* achieved maximum lipid productivity in glycerol supplemented culture medium (Sharma et al., 2016), this could be the reason for the observed increase in lipid content. In addition, the cytosolic lipid bodies increased in number as well as in size as the intensity of the light increased. So, the use of glycerol could advantageous for lipid production.

Tropical microalgae are adapted to high temperatures and sunlight. For this reason, these microalgae can be cultivated outdoors efficiently for lipid production. Wang et al. (2013) showed that lipid and protein content varied with different nitrogen and light regimes. Higher light intensities tend to induce the production of more neutral lipids in comparison to polar structural lipids in algae cells. These tropical freshwater microalgal strains have potential biotechnological applications; therefore, further studies are required on them.

## Conclusion

It was observed that the selected cultivation process can affect the productivity of tropical freshwater microalgae isolates, as chloramphenicol can reduce the growth of microalgae when used at the concentration 50 mg l^−1^. However, the effectiveness of lower concentrations on the elimination of bacteria from the cultures remains to be demonstrated. It is still necessary to determine if the observed deleterious effect of high CAP concentration in *Scenedesmus* sp. R3 growth rate is long lasting in the strains when they are cultured subsequently.

Both DMSO–sucrose and glycerol were found to be effective microalgae cryopreservants in most cases, especially with *Scenedesmus* sp. R3.

The lipid content of the algae was found to be significantly affected by the cryopreservation method. Thus, species such as *Chlorella* sp. M2 and *Chlorella* sp. M6 should be cryopreserved with glycerol when they are cultured to obtain a higher lipid gain per cell. With regard to the antibiotics, it is necessary to determine whether the negative effects of DMSO–sucrose on the algal lipid content is temporary or continues in future cultures.

The results highlight the importance of selecting appropriate methods to obtain axenic cultures and their subsequent storage, as these can severely affect the biological performance of different species of tropical freshwater microalgae isolates. Further, whether these effects are long lasting remains to be determined.

## Supplemental Information

10.7717/peerj.5143/supp-1 Supplemental Information 1 Raw data.

## Additional Information and Declarations

### Data Availability

The following information was supplied regarding data availability: The data is available at GenBank, accession numbers MF677855, MF677856, and MF677857.
